# Satisfaction in complete denture wearers with and without adhesives: A randomized, crossover, double-blind clinical trial

**DOI:** 10.4317/jced.54871

**Published:** 2018-06-01

**Authors:** Carlos Torres-Sánchez, Vanessa Montoya-Salazar, Daniel Torres-Lagares, Jose-Luis Gutierrez-Pérez, Emilio Jimenez-Castellanos

**Affiliations:** 1 DDS, Ms, PhD, Researcher, Stomatology and Dentistry Department, University of Seville, Seville, Spain; 2 DDS, Ms, PhD, Professor of Oral Surgery, Master in Oral Surgery, Oral Surgery Department, Dentistry Department, University of Seville, Seville, Spain; 3 DMD, PhD, Master Chief of Oral Surgery, Dentistry Department, University of Seville, Seville, Spain; 4 DMD, PhD, Professor of Prosthodontics, Dentistry Department, University of Seville, Seville, Spain

## Abstract

**Background:**

The purpose of this study was to compare the satisfaction of patients regarding retention, stability and accumulation of particles with a randomized, double-blind crossed method in users with complete dentures with and without adhesive.

**Material and Methods:**

Seventeen edentulous individuals were randomized and received new upper and lower complete dentures. After a period of adaptation, they participated in some masticatory tests and clinical revisions, after use the protheses with and without the use of two denture adhesives: Adhesive A (Fittydent, Fittydent International GmbH) and adhesive B (Corega, GlaxoSmithKline) at 0, 7 and 14 days. Satisfaction was measured immediately after each test through a survey using a VAS scale (0-10) and data were analyzed with McNemar’s test with Bonferroni correction.

**Results:**

The results showed significant differences (*p*<.01) between the study groups with adhesive A - B and the group without adhesive, but no significant differences were found between the two stickers for any of the variables studied.

**Conclusions:**

Complete denture adhesives significantly improved the satisfaction of patients because a better retention, stability and less accumulation of particles of the food substitute between the denture and the mucosa is obtained compared with non-use of complete denture adhesives.

** Key words:**Complete dentures, patient satisfaction, denture adhesives, clinical trials.

## Introduction

There are numerous alternatives of implant treatment for edentulous patients (EP) with good aesthetic and functional results, providing a higher quality of life (1-6), but still, in certain circumstances the indication of a conventional complete denture (CD) as in the case of elderly multimedicated people with tumoral diseases (7,8), with xerostomia, patients with hormonal and neurotransmitter changes and disorders that affect muscle tension such as Parkinson disease, myasthenia gravis, muscular dystrophy, and buccolinguofacial dyskinesia (9-11).

The use of adhesives for complete dentures (CDAs) is relatively widespread among patients with complete denture which often use them without proper prescription by the dentist, causing dissatisfaction, as a result of not following the instructions of indication and use properly (1,7).

Numerous studies have shown that CDAs with proper prescription of the dentist help to improve the retention and stability of well developed (7,8,12-20) CDs improving the quality of life and general health of the EP and his satisfaction with the use of the CDAs (18-24). In this way the patient satisfaction becomes the most decisive factor in the success of CDs (25-27). Among the most common complaints we can cite the lack of retention or stability and accumulation of particles under the denture (7,9,10).

The composition of the insoluble CDAs is a mixture of salts of polymers such as carboxymethyl cellulose (CMC) and polyvinyl ether Methyl Cellulose (PVM-MA) (12,22,27) whose action mechanism is achieved primarily by an increase in the adhesive and cohesive properties, increasing the viscosity between the CDs and oral mucosa, helping to reduce the movement (27-29) of the prosthesis, achieving a better function and masticatory efficiency reflecting greater patient satisfaction (8,12,21,28-30).

Generally dentists evaluate prosthesis using default criteria for the success based on the technique,^30^-^35^ unfortunately these rules usually do not take into account individual needs and attitudes of patients and their expectations regarding CDs (28-30,35).

The objective of this trial was to compare subjectively through a questionnaire, the feeling of retention, stability and accumulation of particles below the denture among patients with CDs without adhesive (WA) and with two CDAs (adhesive A (Fittydent; Fittydent International GmbH, Pinkafeld, Austria) (AA) or adhesive B (Corega, GlaxoSmithKline, Philadelphia, PA) (BA)). The null hypothesis was that the use of CDAs does not increase the patient satisfaction regarding the evaluation of the retention stability and accumulation of particles of the CDs.

## Material and Methods

The study sample was selected among the EP who attended the School of Dentistry at the University of Seville in demand for treatment between 2013 and 2014. The statistical sampling test used was determined by the formula provided by Torres-Sanchez *et al.* with a method error of 95% confidence (36). All patients should be within the following criteria: to be of legal age, to be EP, without active oral diseases, users of upper and lower new conventional CDs placed two months before the study, which had never used CDAs who were not allergic to any of the components of the CDAs used, without systemic compromise and without physical or mental disability. All prostheses were manufactured in the Stomatological Prosthetics Unit of the Faculty of Dentistry at the University of Seville and always by the same dentist and lab technician (8,12) meeting the criteria for Kapur *et al.* (37). The final sample consisted of 17 patients (11 women and 6 men with an average age of 51.41 years (SD 4.6). The clinical trial was conducted following the ethical principles of medical investigation involving human subjects under the Helsinki Declaration of the World Medical Association (http://www.wma.net) and the Spanish Law 14/2007 of July 3rd for Biomedical Research (http://www.boe.es) (12,38). All of the participants were given a detailed explanation about the purpose and process of the study. The Ethics Committee Approval (Court of Ethics at the University of Seville, US, Spain) and the patients approved written consent were obtained (12,38).

-Clinical Procedure 

The upper and lower CDs were manufactured according to the conventional technique (39), (preliminary impressions, functional impressions with peripheral seal, transfers to semi- adjustable articulator, testing of teeth in wax, placement, occlusal adjustment and controls) in their production and placement the same clinician and laboratory technician were always involved (8,12). In no case the trial was performed before the two months of placement of the dentures to ensure correct soft tissue health and proper adaptation to the prosthesis, but without having been over a year since its placement to avoid mismatches as a result of bone resorption processes of the residual alveolar ridges (8,12,40). Kapur criteria were used to evaluate each patient CDs (37). The EP were randomized by order of arrival in the three groups that performed the tests with and without dental adhesives (8,12) (Group 1: n = 6; Group 2: n = 6 and Group 3: n = 5). In each one of them masticatory tests and clinical revisions were performed in different order, randomly with the three grouping variables: without adhesive (WA), with adhesive A (AA) (Fittydent; Fittydent International GmbH) and adhesive B (BA) (Corega; GlaxoSmithKline). In this way the pattern of learning bias was eliminated in the first masticatory test (8,12).

The AA (Fittydent; Fittydent International GmbH) and BA (Corega; GlaxoSmithKline) were placed in similar white boats without identifying mark for the two products, having an investigator to performed the masticatory tests and a different one to perform data collection.

In this way the double blind method was being applied, by ignoring both the clinician and the patient which of the two adhesives was being used. An irreversible hydrocolloid (12) (Orthoprint; Zhermack SpA, Badia Polinesine, Italy) was used as a meal replacement for the masticatory tests featuring dimensional stability and the possibility of a proper mastication in the EP in order to compare the results of the surveys of the masticatory tests. The tablets were made with the irreversible hydrocolloid (Orthoprint; Zhermack SpA) using a plastic matrix of standardized size and shape (20 mm in diameter and 5 mm in width with a weigh of 2.3 grams) (12,41). The CDs were removed and washed with liquid soap with neutral pH (Avena; ISDIN, Barcelona, Spain) and with a denture brush, 1 cm of the adhesive was applied in three areas: front and two back sides of the upper and lower CDs. the bands of the adhesive were measured with a milimetric ruler and excess removed with a scalpel (8,12).

Mastication was performed in a standardized manner until completing 20 masticatory strokes (8,12,41-43), Each EP received 50 ml of water to wash and remove particles (8,12) of irreversible hydrocolloid (Orthoprint; Zhermack SpA, Roma, Italy).

**Figure 1:**
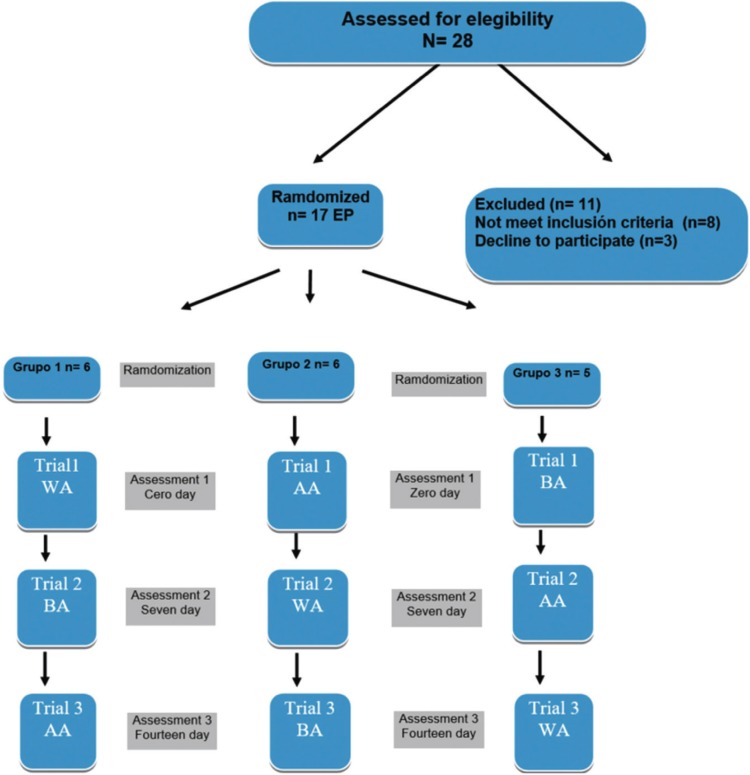
Diagram flow of clinical trials.

The EP carried out the masticatory tests and the satisfaction surveys as follows: day 0 the first test and survey, on day 7 the second test and survey and on day 14 the third test and survey (Fig. 1) (8,12,25). In this way all the EPs performed the masticatory tests and corresponding questionnaires (42,43), rotating by three variables: WA, AA (Fittydent; Fittydent International GmbH, Pinkafeld, Austria) and BA (Corega; GlaxoSmithKline, Madrid, Spain), in different order according to each of the study subgroups.

The questionnaire had the three questions asked to each patient: 1) Lack of retention? (if the patient subjectively felt displacement of the denture due to vertical forces). 2) Lack of stability? (if the patient subjectively felt lateral movement of the denture due to lateral forces). 3) ¿Accumulation of particles? (If the patients felt the accumulation of particles of the substitute between the denture and mucosa). For the objectification of the answers to each of the questions relating to retention, stability and accumulation of particles under the denture, each patient was presented with a VAS scale (0-10), considering as a positive response when the patient marked 7 points or above 7 points and negative when 3 points or below 3 points were marked. (Responses between 3 and 7 were not considered).

-Statistical analyzes 

The grouping variables of the study were WA, AA (Fittydent; Fittydent International GmbH) BA (Corega; GlaxoSmithKline). The results obtained, corresponding to the responses to the survey questions were collected in a file of IBM SPSS Statistics 22 (licensed from the University of Seville) (IBM, New York, USA), for appropriate processing (7,12).

## Results

The survey data and the subjective evaluation after masticatory tests by the EP with respect to retention, stability and accumulation of particles for the three studied groups (WA, AA (Fittydent were collected, Fittydent International GmbH) and BA (Corega GlaxoSmithKline)) was presented in  Table 1.

**Table 1:**
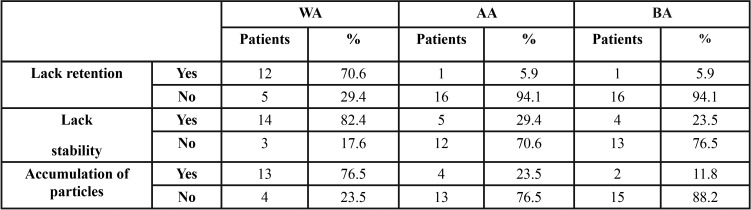
Answer for question of patient satisfaction. Statistically significant differences between all the WA and AA groups’ data and all the WA and BA groups’ data.

It is observed how for the three variables, retention, stability and accumulation of particles, the percentage of “Yes” and “No” are very similar inverted in the case of the use of adhesive A and B (Lack of retention with AA: Yes 1 - 5.9%, No 16 - 94.1%; Lack of retention with BA: Yes 1 - 5.9%, No 16 - 94.1%), (Lack of stability wth AA: Yes 5 - 29.4%, No 12 - 70.6%; Lack of stability with BA: Yes 4 - 23.5 %; No 13 - 76.5%), (Accumulation of particles with AA: Yes 4 - 23.5%, No 13 - 76.5%; Accumulation of particles with BA: Yes 2 - 11.8%, No 15 - 88.2%).

Compared to the values in the tests without the CDAs (Lack of retention WA: Yes 12 -70.6%, No 5 - 29.4%; Lack of stability WA: Yes 14 - 82.4%, No 3 - 17.6%; Accumulation of particles WA: Yes 13 - 76.5%, No 4 - 23.5%) those data are much better.

The inferential analysis was planned to compare the variables Retention / Stability / Accumulation of particles among the three groups using the Cochran test which resulted statistically significant (*p* <0.01) 

To establish which particular groups appear among the above differences in each of the studied variables, pairwise comparisons were established by the McNemar test with Bonferroni correction. The result for each of the variables studied was the same, namely, there were significant differences (*p* <0.01) between the study groups AA or BA and the group WA, but there were no significant differences between both groups of AA and BA together for any of the variables studied.

## Discussion

The results of this clinical trial show that there is a significantly higher patient satisfaction when using the CDAs on their CDs. This is mainly because the CDAs through their composition with (CMC) and (PVM-MA) have an action mechanism that achieves an increase in adhesive and cohesive properties, increasing the viscosity between CDs and the oral mucosa. In this way the CDAs used contribute to the reduction of movement (8,12,22,25) of the CDs achieving a better function and masticatory efficiency which reflects higher patient satisfaction (8,12,22).

The results of this study are consistent with those of other authors (12,28-30,41,42) who found that CDAs significantly reduced the movement of the maxillary and mandibular denture during mastication and increased comfort resembling dentate patients who do not use CDs. In addition, patients reported that the use of CDAs avoided the inconveniences caused by food particles that are introduced below during mastication, causing irritation and pain in the mucosa due to friction.

Most authors show a recognized secondary benefit of CDAs in patients with CD, which properly used have the ability to act as a barrier to help prevent the migration of food particles under themselves. Unfortunately this could not be measured in an objective way to show numerical results (30,34,35,37). In this clinical trial VAS scales have been used to quantify the variables. We decided to treat them as qualitative variables, though with a 4-point objective differentiation between them (Yes: above 7 points; No: below 3). In this way greater rigor is achieved, since it would have been easier to obtain statistically significant differences with quantitative variables, although they were clinically irrelevant.

The results of this study are consistent with those of Kawata *et al.* (43) They find that the subjective feeling of patients is of a greater comfort by feeling less lateral movement and less displacement from their CDs during the masticatory tests and that this reflected less fatigue in their muscles by the end of each masticatory test, coinciding with studies where muscle fatigue due to overload decreases as the oral cavity has higher masticatory efficacy (22). Regarding the success of the CDs measured by surveys, the results of De Lucena *et al.* (28) and Celebic *et al.* (29) revealed that 39% of the volunteers were extremely dissatisfied with their dentures, contrary to the values reported by other studies (33,34).

On the other hand, a surprising finding was the large number of very satisfied volunteers despite their CDs were quite old and mismatched. When the results of patient satisfaction and the functional evaluation of the dentures made by the clinician were correlated, no significant correlations between the two assessments were observed. Similar findings have been previously reported by other authors (32-35), suggesting that, while important, technical manufacturing aspects of the CDs are not sufficient to predict the success of treatment from the standpoint of patients.

Van Waas (32) studied a group of patients using new CDs, finding that only 13% of those who had mentioned being satisfied with their prostheses coincided with the favorable assessment by the investigator.

In this regard, it has been suggested that other factors such as attitude towards CDs, number of CDs used previously, personality of patients, expectations, patient-dentist relationship and even judgment of qualifications of the dentists and their skills can play an important role in the final assessment of patient satisfaction regarding treatment (32-35,42). Dentists often evaluate prosthesis using defaults for success based on technical criteria, rules usually do not take into account individual needs and attitudes of patients or their expectations of CDs (35).

## Conclusions

According to the results obtained, with the logical limitations of this study and in response to the objectives, we can make the following conclusions: 1) The CDAs significantly improved the satisfaction of EP because a better retention stability and accumulation of food substitute between the denture and mucosa was obtained compared with not using CDAs. 2) No significant differences exist in the satisfaction of EP in terms of retention, stability and accumulation of particles of food substitute between the denture and the mucosa when using the two CDAs of the clinical trial.
